# Development of Interoperable Computable Phenotype Algorithms for Adverse Events of Special Interest to Be Used for Biologics Safety Surveillance: Validation Study

**DOI:** 10.2196/49811

**Published:** 2024-07-15

**Authors:** Ashley A Holdefer, Jeno Pizarro, Patrick Saunders-Hastings, Jeffrey Beers, Arianna Sang, Aaron Zachary Hettinger, Joseph Blumenthal, Erik Martinez, Lance Daniel Jones, Matthew Deady, Hussein Ezzeldin, Steven A Anderson

**Affiliations:** 1 IBM Consulting Bethesda, MD United States; 2 Accenture Inc Ottawa, ON Canada; 3 Center for Biostatistics, Informatics and Data Science MedStar Health Research Institute Columbia, MD United States; 4 Department of Emergency Medicine Georgetown University School of Medicine Washington, DC United States; 5 Center for Biologics Evaluation and Research United States Food and Drug Administration Silver Spring, MD United States

**Keywords:** adverse event, vaccine safety, computable phenotype, postmarket surveillance system, real-world data, validation study, Food and Drug Administration, electronic health records, COVID-19 vaccine

## Abstract

**Background:**

Adverse events associated with vaccination have been evaluated by epidemiological studies and more recently have gained additional attention with the emergency use authorization of several COVID-19 vaccines. As part of its responsibility to conduct postmarket surveillance, the US Food and Drug Administration continues to monitor several adverse events of special interest (AESIs) to ensure vaccine safety, including for COVID-19.

**Objective:**

This study is part of the Biologics Effectiveness and Safety Initiative, which aims to improve the Food and Drug Administration’s postmarket surveillance capabilities while minimizing public burden. This study aimed to enhance active surveillance efforts through a rules-based, computable phenotype algorithm to identify 5 AESIs being monitored by the Center for Disease Control and Prevention for COVID-19 or other vaccines: anaphylaxis, Guillain-Barré syndrome, myocarditis/pericarditis, thrombosis with thrombocytopenia syndrome, and febrile seizure. This study examined whether these phenotypes have sufficiently high positive predictive value (PPV) to ensure that the cases selected for surveillance are reasonably likely to be a postbiologic adverse event. This allows patient privacy, and security concerns for the data sharing of patients who had nonadverse events can be properly accounted for when evaluating the cost-benefit aspect of our approach.

**Methods:**

AESI phenotype algorithms were developed to apply to electronic health record data at health provider organizations across the country by querying for standard and interoperable codes. The codes queried in the rules represent symptoms, diagnoses, or treatments of the AESI sourced from published case definitions and input from clinicians. To validate the performance of the algorithms, we applied them to electronic health record data from a US academic health system and provided a sample of cases for clinicians to evaluate. Performance was assessed using PPV.

**Results:**

With a PPV of 93.3%, our anaphylaxis algorithm performed the best. The PPVs for our febrile seizure, myocarditis/pericarditis, thrombocytopenia syndrome, and Guillain-Barré syndrome algorithms were 89%, 83.5%, 70.2%, and 47.2%, respectively.

**Conclusions:**

Given our algorithm design and performance, our results support continued research into using interoperable algorithms for widespread AESI postmarket detection.

## Introduction

### Background

The US Food and Drug Administration (FDA) Center for Biologics Evaluation and Research (CBER) is responsible for ensuring the safety, purity, potency, and effectiveness of biological products. This includes vaccines; allergenics; blood and blood products; and cell, tissue, and gene therapies for the prevention, diagnosis, and treatment of human diseases, conditions, or injuries [1]. The FDA’s history of safety surveillance for vaccines includes the creation and monitoring of the Vaccine Adverse Event Reporting System (VAERS). VAERS, jointly administered by the FDA and the Centers for Disease Control and Prevention (CDC), accepts spontaneous reports of suspected vaccine adverse events (AEs) after administration of any vaccine licensed in the United States.

VAERS has been successfully used as an early warning system to identify rare AEs; however, it has limitations. VAERS is a passive surveillance system that relies on individuals, patients, and clinical staff to send in reports, as opposed to automatically collecting them based on clinical data. This can lead to undercounting AEs. In addition, a causal relationship cannot be established using information from VAERS reports alone [2]. Because of VAERS’s limitations, more robust data systems are needed to enhance AE detection. These systems would be especially important for detecting the most severe AEs that require medical attention so that the FDA and CDC can offer guidance for these potentially life-threatening events and ensure that product labeling reflects known risks.

To address this gap, CBER established the Biologics Effectiveness and Safety Initiative (BEST) Initiative in 2017 to build data assets, analytics, and infrastructure for an active, large-scale, efficient postmarket surveillance system that can evaluate the safety and effectiveness of biologic products and develop innovative methods [3]. The BEST system is a collection of real-world data (RWD) sources: data related to patient health status and the delivery of health care that are routinely collected from several sources, such as electronic health record (EHR) or claims data [4]. EHR databases, specifically, are a rich source of information. They include data such as clinical notes, which can help address the limitations of VAERS. They also include entire populations of patients to identify whether cases are underreported. In addition, they may include patients’ entire clinical history, which can help establish a causal relationship for an AE. BEST has reached agreements with a limited number of foundational data partners. Access to these data partnerships does not fully address the possible undercounting of AEs of special interest (AESIs). However, these partnerships allow accelerated development and testing of AESI detection algorithms.

BEST is currently researching a system of distributed computable phenotype algorithms that could be applied at scale to many or all EHR systems across the United States to semiautomatically detect and report potential AESIs from RWD. Such a system could increase the speed and scope of AE surveillance beyond what is currently available to public health agencies through data partner agreements. To be candidate phenotypes for distributed surveillance use, the phenotypes need to identify probable AEs and avoid false detections. This reflects the need to balance the correct detection of AESIs with the protection of privacy and the reduction of burden on health provider systems. For the wider population of health providers to consider deploying such detection algorithms, these phenotype algorithms need to have reasonably high performance (measured by positive predictive value [PPV]) to ensure that the cases identified as AEs are likely to be verifiable cases with the outcome of interest. Toward this goal, the computable phenotypes in this study focus on existing EHR data reflecting a detected AE, which are reportable events for public health purposes. The algorithmic identification of undetected AEs or AEs that were not coded properly is beyond the scope of this study. Such research must include data from patients who had no AEs to fully evaluate the performance of a computable phenotype algorithm. Although scientifically desirable in the long term, the inclusion of non-AE cases falls outside of initial goals for a distributed surveillance system, which is assessing performance (measured by PPV) of the phenotypes for wide-scale surveillance purposes. The goal of distributing the phenotypes also poses limitations on designing the algorithms. Specifically, the components and complexity of the underlying algorithms need to take into account the current EHR standards and technology because they must be deployable and executable across EHR databases without imposing large overhead on health provider systems. If the phenotypes have sufficient PPV and are sufficiently easy to implement at health provider sites, the FDA could share the phenotypes to detect AESIs following vaccination in EHRs across the country, which could then be reported to the FDA for further review. The ability to detect AESIs using RWD could create an active surveillance system that enhances overall vaccine safety and helps make recommendations to minimize risks for postvaccination AESIs. The implementation of algorithmic detection and automated reporting of AESIs found in RWD has been shown to increase the odds of submitting a VAERS report by >30 times the preimplementation rate [5].

### Objective

Although there is a history of studies around postvaccination AESIs, including those for influenza [6-8] and COVID-19 vaccines [9-13], there has been an increased interest in the analysis of vaccine safety and surveillance since the emergency use authorization (EUA) of 3 COVID-19 vaccines in the United States (Pfizer-BioNTech, Moderna, and Novavax) and their subsequent boosters (eg, bivalent boosters). The FDA hopes to contribute to this research through the development and performance validation of phenotypes for 5 postvaccination AESIs to identify potential vaccine safety events within EHR databases for this study. The 5 AESIs chosen include myocarditis/pericarditis, anaphylaxis, Guillain-Barré syndrome (GBS), intracranial or intra-abdominal thrombosis with thrombosis with thrombocytopenia syndrome (TTS), and febrile seizure. These AESIs were chosen because they are documented priorities of the CDC’s vaccine surveillance [14] for COVID-19 vaccine safety. In addition, several of these AESIs (anaphylaxis, GBS, and febrile seizure) are found following exposure to other vaccines, such as influenza; shingles; pneumococcal conjugate; and measles, mumps, and rubella. This study describes the methods to develop and validate these 5 computable phenotype algorithms on an EHR database and the validation results. It is part of the FDA’s efforts to improve postmarket surveillance and is valuable for public awareness, safety, and transparency.

## Methods

### Ethical Considerations

Ethical approval was not required for the study involving humans in accordance with the local legislation and institutional requirements. This study was part of the Sentinel activities conducted by the FDA as part of its postmarket surveillance duties. The Office of Human Research Protection (OHRP) in Health and Human Services (HHS) determined that the studies done under the Sentinel programs are not subject to regulation (45 CFR part 46) administered by OHRP. Written informed consent to participate in this study was not required from the participants or the participants’ legal guardians or next of kin in accordance with the national legislation and the institutional requirements.

### Computable Phenotype Development

#### Overview

In total, 5 AESIs were selected to develop computable phenotypes for our validation study. The study’s main focus was detecting COVID-19 vaccine–related AESIs; therefore, we selected AESIs that the CDC specifically identified for monitoring after COVID-19 vaccination [14] or AESIs that have been reported for some subpopulations [15]. Given the uncertainty about the future use of COVID-19 seasonal boosters, the FDA also wanted to ensure that the AESIs selected had broad applicability to the safety surveillance of other widely used vaccines such as influenza; shingles; pneumococcal conjugate; diphtheria-tetanus-pertussis; and measles, mumps, and rubella. Three of our 5 selections met those criteria given in the CDC’s documented monitoring of anaphylaxis [16], GBS [17], and febrile seizures [18] for at least one of the vaccines listed.

The phenotype algorithms were designed to be relatively simple and interoperable so that any new health care organization’s IT department could translate and run them on their EHR database. They were built to query only structured data for interoperable, standard codes, such as Logical Observation Identifiers Names and Codes, Systematized Nomenclature of Medicine Clinical Terms, and RxNorm, so that the algorithm can be generalized or translated across different EHR systems. Historically, this has been a challenge for developing algorithms, since EHR databases often contain their own local code systems specific to the EHR vendor. For example, for this effort, we worked with the study partner to map Cerner Multum medication and observation codes to standard RxNorm and Logical Observation Identifiers Names and Codes, respectively.

Recent regulation now requires each EHR database to have an application programming interface (API) endpoint that translates any EHR data and many of the EHR’s proprietary codes to the United States Core Data for Interoperability (USCDI) implementation of the Fast Healthcare Interoperable Resources (FHIR) specification [19]. This specification requires the use of interoperable, published code lists [20] (Table 1). These code systems cover almost all clinical events for the detection of AEs, such as medical diagnoses, medication prescriptions, laboratory tests or vital signs taken, and procedures performed. These APIs currently focus on supporting use cases where a single patient’s data are queried as opposed to aggregate searches across patients; therefore, we were unable to use them to identify the cohort that our phenotype would select. We were, however, able to use the FHIR API endpoints to pull data for each patient in our validation samples so that the participating clinicians could have data with the standard, interoperable code sets for their review.

**Table 1:** AESI^a^ case definitions and descriptions.

AESI	Description	Case definition reference
Myocarditis/pericarditis	Myocarditis and pericarditis are inflammatory processes involving the myocardium, pericardium, or both (myopericarditis).	Morgan et al [21], 2008
Anaphylaxis	Anaphylaxis is an acute hypersensitivity reaction with multiorgan system involvement that can present as, or rapidly progress to, a life-threatening reaction. It may occur following exposure to allergens from a variety of sources, including food, aeroallergens, insect venom, drugs, and immunizations.	Rüggeberg et al [22], 2007
GBS^b^	GBS constitutes an important proportion of acute flaccid paralysis cases worldwide. It is a condition characterized by various degrees of weakness, sensory abnormalities, and autonomic dysfunction due to damage to peripheral nerves and nerve roots.	Sejvar et al [23], 2011
Intracranial or intra-abdominal TTS^c^	Several cases of unusual thrombotic events and thrombocytopenia have developed after vaccination with the recombinant adenoviral vector encoding the spike protein antigen of SARS-CoV-2 (ChAdOx1 nCov-19, Astra Zeneca). More data were needed on the pathogenesis of this unusual clotting disorder [24].	Chen and Buttery Monashm [25], 2021
Febrile seizure	There is no Brighton Collaboration definition of febrile seizure, so we used both the fever and seizure case definitions. *Fever* is defined as an elevation of body temperature above normal. It is usually caused by infection but can also be associated with several immunologic, neoplastic, hereditary, metabolic, and toxic conditions. Seizures are episodes of neuronal hyperactivity, most commonly resulting in sudden, involuntary muscular contractions.	Marcy et al [26], 2004; Bonhoeffer et al [27], 2004

^a^AESI: adverse event of special interest.

^b^GBS: Guillain-Barré syndrome.

^c^TTS: thrombosis with thrombocytopenia syndrome.

**Figure 1:**
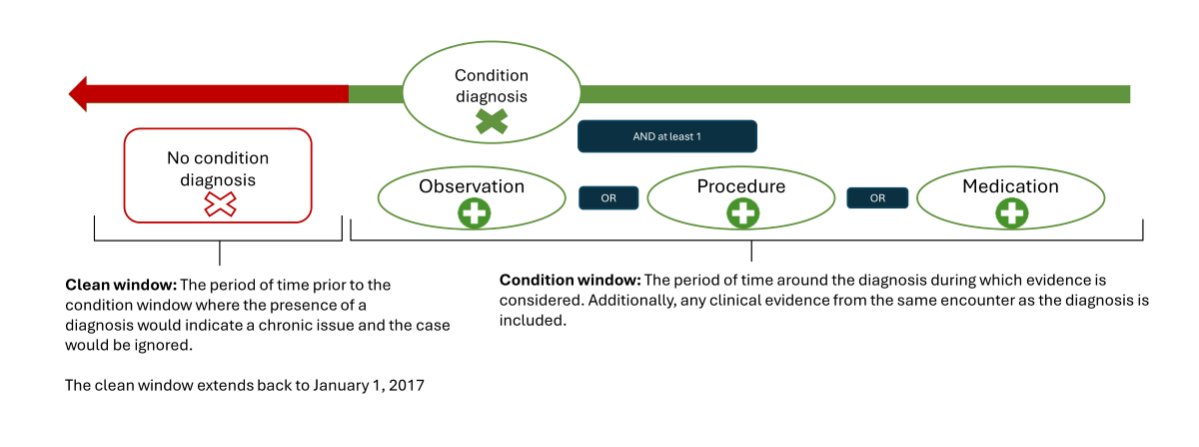
Composition algorithm modified for each adverse event.

To facilitate health provider organizations’ ability to implement these queries on their EHR, phenotypes were rules based, used only certain types of structured data, and used common logic across AESIs. The general phenotype logic has been used previously for several postvaccination AESI studies at the FDA to identify potential AESI cases [28,29] and reuses concepts and methods from past literature from US-based collaborative health research groups, such as Observational Health Data Sciences and Informatics (OHDSI) [30], or from similar efforts in the United Kingdom [31] to develop computable phenotype libraries. A health organization only needs to write the general query logic once and then this logic would be able to detect different types of AESIs by referencing different lists of medical codes that represent the different medical events providing evidence that the various AESIs occurred. The logic common to all phenotypes is shown in Figure 1. The code lists that we developed for necessary types of medical evidence are described in more detail below and listed in Table S1 in Multimedia Appendix 1 [21-23,25-27]. The circled items in Figure 1 represent a search for an FHIR resource element containing a code in one of the developed code lists. These were applied within the windows of time denoted by the brackets identifying windows of time before and after a condition diagnosis. The concepts in Figure 1 are described in additional detail below.

#### AESI Diagnoses and Problem List Items

The algorithm first looks for evidence of the AESI represented by a coded final or discharge diagnosis. Only final or discharge diagnoses are used since they best represent the ultimate determination of what was diagnosed during the patient’s care. The variability of admitting, working, and other diagnosis types lack the specificity required for the algorithm in this study.

#### Care Setting Filters

In addition, the care setting for every diagnosis was collected based on the medical encounter type for the diagnosis. All diagnosis care settings values were grouped into inpatient, outpatient, or emergency care setting types. Care setting was used to filter out diagnosis codes made during encounters with care settings unlikely to have the specific AESI diagnosis in the phenotype. The included care settings are defined by case definition and clinician input.

#### Clean Window

Next, a clean window (ie, a period before the coded diagnosis identified in step 1) is checked to ensure that the target diagnosis is the first known diagnosis of its type. This prevents the inclusion of historical or ongoing conditions. For all algorithms in this paper, the clean window is defined by all historical patient data in our data set. To make sure that all patient cases had at least a 1-year clean window, we pulled an additional historical year of data from our data partner before the study period. Cases where there were multiple occurrences of an AESI diagnosis suggested possible evidence of a chronic condition unrelated to vaccine exposure and thus were excluded.

#### Condition Window

Finally, the algorithm searches for sufficient supportive evidence within a condition window. The *condition window* is defined around the AESI diagnosis date and includes the entire medical encounter period when the condition was diagnosed, as well as 2 days before and 10 days after a condition is diagnosed. Clinical subject matter experts defined condition windows as the timeframe around a diagnosis that supportive evidence would likely present itself in the medical record.

#### Supporting Evidence

Within the condition window period, the algorithm may filter cases based on supporting evidence of an AESI. This filter looks for either laboratory test results found in observations, AESI treatment procedures, AESI treatment medications, or procedure or a combination of the 3 supporting evidence with a code that matches a code on to the phenotypes’ concepts code lists. These code lists in the lists aim to include all medical codes that could represent a particular concept, such as administration of epinephrine for an anaphylactic reaction. This AESI supporting evidence filter was applied to all phenotypes except for our febrile seizure AESI phenotype because a review of existing research [32] showed febrile seizure algorithms, in general, had the highest PPV among the selected AESIs. The concepts to build code lists for the supporting evidence were identified using case definitions. Following this, we prioritized improving specificity in the other AESI phenotypes by including filters requiring additional supporting evidence [32-35] and our clinician’s input.

#### Vaccine Exposure

In real-world operation, the algorithm would also include a vaccine exposure and risk window or a period surrounding vaccination in which diagnoses are searched. For the study’s purposes of having sufficient volume and statistical power to estimate operating characteristics of the algorithm, these exposure rules were not included.

#### Additional Details

Ideally, to assess whether these algorithms generalize to other sites, we would have a multisite validation study. Because of the high cost of data agreements, however, we only had data available for a single EHR site. To avoid overfitting and ungeneralizable results, we designed our algorithm development methods to only use our EHR data as a validation set and not use any of it to train, develop, or fine-tune the algorithm. While this does not remove the need for additional external validation, it reduces the likelihood of finding ungeneralizable results. To identify what medical concepts the algorithm should use as evidence, clinicians identified observations, medications, conditions, and procedure concepts from the AESI’s case definition, their relevant clinical experience, or other research from their literature review. A brief description of the AESI and the reference of the case definition used is captured in Table 1, and additional information on the case definition is saved in Table S2 in Multimedia Appendix 1 [21-23,25-27].

An analyst completed a text search for a list of terms for these identified concepts, a list of which is captured in Table S2 in Multimedia Appendix 1, to build the code lists of relevant codes from selected interoperable coding libraries (Textbox 1) [21-23,25-27]. This was accomplished by searching the open-sourced OHDSI Observational Medical Outcomes Partnership concepts table and ATLAS tool (OHDSI community) [36], which is a collection of thousands of interoperable codes and their definitions and descriptions. The table was searched for any definition or description that matched the identified concept for the interoperable code systems that we listed in Textbox 1 and then was reviewed by a clinician for their suitability for the algorithm.

**Codes used for each type of clinical data.**

**Clinical data and interoperable code lists used**

- Diagnosis: *International Classification of Diseases, Tenth Revision, Clinical Modification*, Systematized Nomenclature of Medicine Clinical Terms
- Medication or immunization: National Drug Code, RxNorm
- Procedures: Current Procedural Terminology, *International Classification of Diseases, 10th Revision* Procedure Coding System
- Observations: Logical Observation Identifiers Names and Codes

The immunization and the diagnosis *International Classification of Diseases, 10th Revision*, *clinical modification* (*ICD-10*) and Systematized Nomenclature of Medicine Clinical Terms code lists have been published on the Value Set Authority Center [37], and the additional observation, medication, and evidence code lists may be added in the future after this study is published.

For a surveillance use case, the algorithms need be run regularly (eg, daily or weekly) to collect batches of historical cases once all the data are available (as opposed to a real-time implementation to collect cases as they are happening). Because the algorithms were created to prioritize simplicity and interoperability rather than maximize total performance (eg, metrics beyond PPV such as sensitivity and, negative predictive power, etc), this study aimed for improved performance (measured by PPV) to existing AESI claims-based algorithms. Given our knowledge of how some crucial distinguishing information is part of unstructured clinical notes, which are not considered by the algorithms in this study, we expect further analysis is needed to improve accuracy [38,39]. Natural language processing techniques can improve algorithm performance but greatly increase the deployment complexity across health care organizations. Therefore, no natural language processing techniques were used for any phenotypes designed for this study.

### Study Period

The study period spanned from January 1, 2018, through May 1, 2022, to ensure that the study’s data sampled patients both before and after the FDA issued the EUA and full licensure for COVID-19 vaccines. We also pulled at least 1 year of historical data for all patients; therefore, our data set includes historical information from January 1, 2017, to January 1, 2018, for all patients with medical encounters in the study period. Patients were included even if there were no clinical events in their historical period.

### Data

The study population came from a single academic health system in the United States, with EHR medical encounter data from >2.6 million patients and >20.7 million medical encounters for the study period. Table 2 shows the demographic breakdown for age, gender, race, and ethnicity of this population.

The entire EHR population during the study period was eligible to be selected by one of our developed phenotype algorithms. There were no age-related, medical condition–related, or other exclusions on the population for the algorithm to select cases. Clinical data necessary to select and validate cases selected by the algorithm were provided to the study team through a series of EHR data extracts for all patients in the study period. The algorithm required the following clinical data categories:

- demographic
- encounter
- condition
- procedure
- medication
- observation

**Table 2:** Demographics of academic health system for study population (N=2,666,974).

Category and demographic group			Patients, n (%)	
**Age (y)**				
	<5	96,146 (3.6)		
	5-17	224,941 (8.4)		
	18-24	224,631 (8.4)		
	25-44	840,395 (31.5)		
	45-64	689,075 (25.8)		
	≥65	591,497 (22.2)		
	Missing	289 (0.01)		
**Sex**				
	Male	1,167,374 (43.8)		
	Female	1,494,096 (56.1)		
	Missing	5504 (0.2)		
**Race**				
	Black or African American	748,746 (28.1)		
	American Indian or Alaska Native	5834 (0.2)		
	Asian or Pacific Islander	53,666 (2)		
	White	1,030,834 (38.7)		
	Other	198,265 (7.4)		
	Unknown	629,608 (23.6)		
	Declined to answer	21 (0)		
**Ethnicity**				
	Hispanic	94,207 (3.5)		
	Non-Hispanic	1,866,561 (70)		
	Unknown	706,206 (26.5)		

EHR data extracts were mapped and loaded into an OHDSI Observational Medical Outcomes Partnership database [40]. Medication, observation, and procedure data extracts were requested and loaded into the database only for patients who would not be disqualified by other algorithm criteria. For patients selected to be in the validation sample, these data along with the clinical data for allergies, immunizations, and clinical notes were pulled from the EHR’s FHIR API endpoints, patient by patient, using a custom Python script to loop through the patients in the sample. The data were loaded into a Health Level 7 API (HAPI) FHIR server. We only pulled FHIR data for cases not initially disqualified by the vaccination and diagnosis filters to avoid unnecessary large data transfers and storage. The algorithm flagged potential AESIs that met the specified criteria. Samples of these cases were sent to physicians for validation.

### Validation Sample

**Figure 2:**
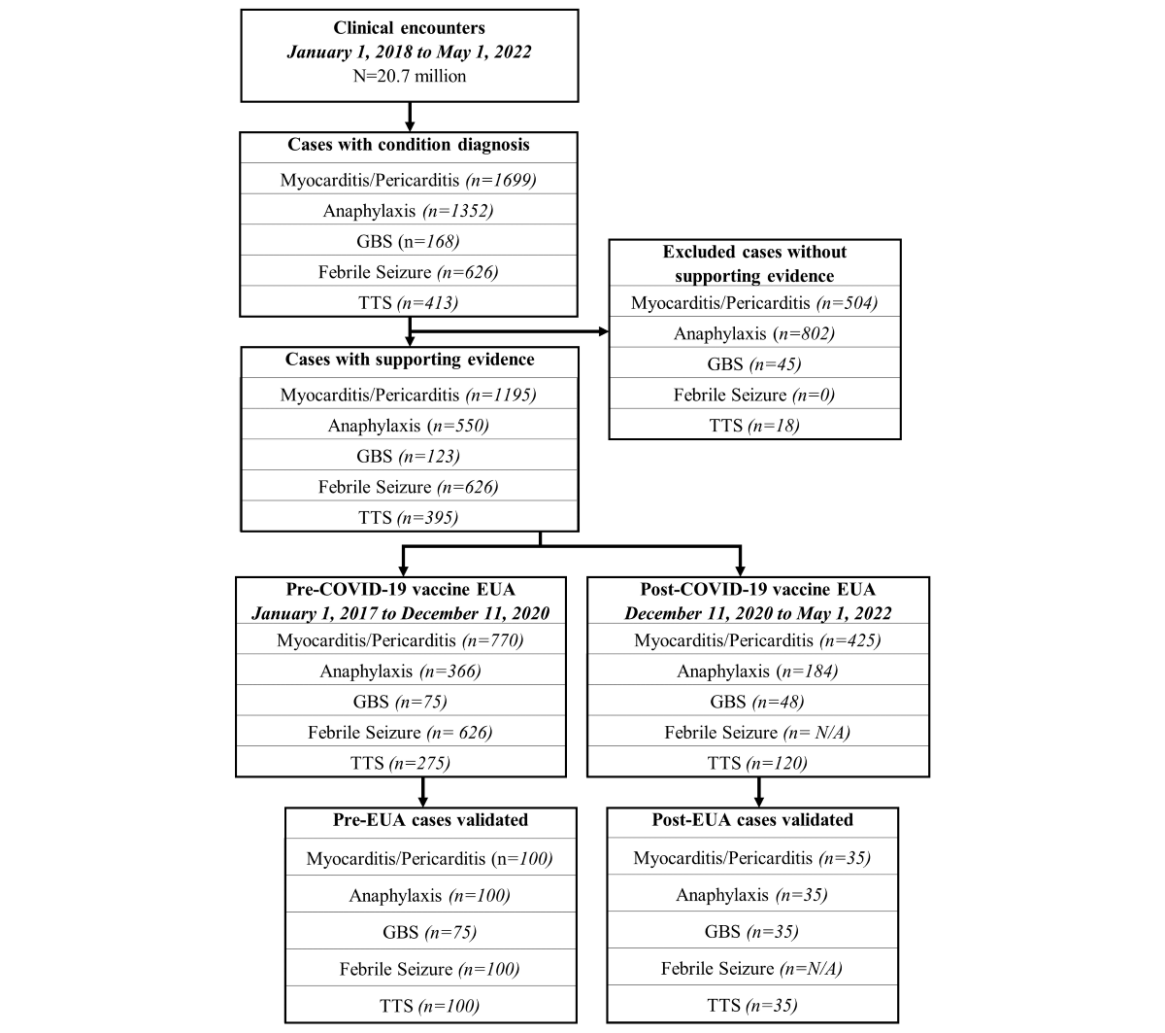
Study population CONSORT (Consolidated Standards of Reporting Trials) diagram. EUA: Emergency Use Authorization; GBS: Guillain-Barré syndrome; N/A: not applicable; TTS: thrombosis with thrombocytopenia syndrome.

Once the algorithm identified cases, a random sample was drawn for each AESI for clinician adjudication. We used stratified sampling to ensure cases during pre– and post–COVID-19 EUA periods were represented (Figure 2). This was due to concerns regarding potential confounding introduced by the COVID-19 vaccines, when attention to possible AESIs or medical charting of AESIs may have shifted. Where possible for each AESI, 100 cases were sampled from the pre–COVID-19 EUA period and 35 from the post–COVID-19 EUA period. If there were <100 or <35 cases during these periods, respectively, the sample would contain all cases the algorithm selected. Febrile seizure was the exception, as we believe the COVID-19 vaccine EUA should not affect the algorithm’s performance because febrile seizure AEs are usually associated with pediatric populations, and the COVID-19 vaccine was not approved for these populations during the study period [27].

Case counts sampled in each period were based on the incidence of diagnosis code occurrence within each period, as well as the period covered. In addition, we added negative controls selected randomly from every encounter in the period to establish a baseline comparison for the case validation process. We included negative controls as a quality control step to reduce the chance of quality issues with the data and to review the methods our clinicians were following and not for the purpose of making inferences about the phenotypes’ performance for non-AE cases (eg, through metrics such as sensitivity, negative predictive power, or an overall metric for performance). This study did not focus on the algorithmic identification of undetected AEs or AEs that were not coded properly. The focus of this study was to determine the phenotypes’ PPV. Given the expense of clinicians’ time for validations and the rarity of the AESIs, there would be minimum benefit to this study to have a negative control sample large enough to draw strong inferences. Furthermore, negative case controls would not further validate the utility of the phenotypes as tools for identifying probable AESIs through distributed surveillance. We added 20 negative controls from the pre–COVID-19 EUA period and 7 from the post–COVID-19 EUA period. Physicians were blinded to which cases were controls and which were not.

### Chart Review Process

The sample of cases used to validate the algorithm was loaded into a chart review tool for clinician review. This allowed the clinicians to sort through the clinical information for a case and record the determination. Each case was assigned to 2 clinicians for review. The clinical validation used a patient’s full clinical history, which included EHR data, including all clinical notes for each case. The full EHR data used for clinician review included data unused by the detection algorithm described in the Computable Phenotype Development section, including different types of data (eg, allergies and clinical notes) and data filtered out (eg, admitting diagnosis and encounters with different care settings).

For each case, the clinician evaluated whether the clinical data evidence met the specified case definition criteria. Relevant patient data for the case window were available and presented to the clinicians in an easy-to-use, browser-based tool with a custom user interface. In the tool, clinicians were able to group items by type, search across all items and text, and request additional chart data to expand the window and access any available historical patient data, if desired.

All suspected AEs were validated using published case definitions [21-23,25-27] according to the levels of diagnostic certainty: level 1 (definite), level 2 (probable), and level 3 (possible). If a case did not meet one of the levels in the case definition, it was assigned as level 4 (doubtful) or level 5 (ruled out). “Ruled out” is distinct from “doubtful” in that “ruled out” cases have definitive evidence disqualifying them from being a correct diagnosis. If a case was determined to be “definite” or “probable,” it was considered a positive case of the AESI.

In the event of a disagreement between a positive and negative clinical review, a third clinician made a final determination by reviewing the case EHR data. If the clinicians found the structured or unstructured EHR data was insufficient, they marked this in their review by creating a level 3 (possible, insufficient evidence) designation, where an AESI could have occurred, but where there was not enough documentation to fulfill the requirements of the case definition.

### Statistical Analysis

#### PPV of Algorithms

Each algorithm’s PPV was the proportion of positive AEs the algorithm identified that were confirmed by clinical adjudication. PPVs were calculated for each AESI overall, as well as stratified by pre– and post–COVID-19 EUA periods and care setting (inpatient, emergency department, or outpatient). Sensitivity analyses were performed to evaluate the impact of medication use, different case definitions, and levels of evidence. PPVs were calculated in 2 different ways for each AESI algorithm. The first PPV calculated removed all possible cases with insufficient evidence from the denominator (cases labeled “definite” and “probable”/total cases minus any labeled “possible, insufficient evidence” by clinicians). PPV was then calculated with the cases with insufficient evidence added back into the denominator (cases labeled “definite” and “probable”/total cases). Reporting both PPV calculations can help with understanding the performance for different algorithm uses. Algorithm performance should ideally be compared with past literature of detection algorithms for the same AESI.

#### CI Values

Because PPV is a binominal proportion, we calculated CIs for the PPV using the Agresti-Coull interval [41], which is the recommended method for estimating accurate CIs for binomial proportions such as PPV [42].

#### Interrater Reliability

Interrater reliability was used to measure the extent to which 2 physicians agreed in their AESI assessment. It was calculated using Cohen κ between the first 2 reviewers. Cohen κ measures the agreement between 2 raters classifying instances into mutually exclusive groups [43].

### Stratification Analysis and Sensitivity Analysis

After validation was completed, we conducted a stratification and sensitivity analysis. We selected 2 stratification variables that could reasonably impact the generalizability of the results. First, we stratified the data by pre- and post-EUA date to confirm that the algorithm behavior did not change for AESIs after the COVID-19 vaccine was approved and administered to a large portion of the population. Ideally, the algorithms would perform consistently across these eras, but there are multiple factors that could impact the performance over these time periods. We also stratified the data by the care setting of the AE diagnosis, given that care settings may be associated with varying EHR data elements (eg, emergency departments compared with inpatient settings). Algorithm performance was computed using PPV within each stratum.

We also completed a post hoc sensitivity analysis where we investigated whether the algorithm could be improved, as measured by PPV, through small changes to it or by updating the process for evaluation. These changes were based on insights from clinicians or data analysts reviewing validation results, so results may not generalize to other data sets. However, we did attempt to limit our analysis to decisions that could have been feasibly made without postvalidation insights. The changes to the algorithms were either removing medications, observations, procedures, or diagnosis codes that are not specific enough to the AESI in question or adding logic to further filter out cases by requiring more supporting evidence (Table 3).

The stratification or sensitivity analyses are meant as exploratory analyses to prompt additional research, but subgroups often have too small a sample size that have narrow enough CIs for meaningful results.

We also completed a sensitivity analysis on the GBS algorithm to calculate the PPV if we relaxed some of the specific case definition evaluation criteria and if more general evidence was available. We found that the 2 pieces of evidence that the case definition required were often missing in the chart review tool: lack of cerebrospinal fluid (CSF) white blood cell (WBC) count in cases of elevated CSF protein and limited or inconsistent documentation of diminished or absent reflexes. In some of these cases, we saw evidence that a neurologist was consulted and felt there was strong suspicion of GBS despite the missing documentation for these tests. This could be explained by 2 mechanisms.

First, and most likely, this could be due to data loss during the delivery or translation of EHR data to our chart review tool. Because we did not have direct access to the data, our process for obtaining, translating to different common data models or standards, and presenting the data to clinicians using the chart review tool could cause the data for these tests to be incorrectly mapped.

**Table 3:** Total list of all sensitivity analyses for each adverse event of special interest (AESI).

AESI	Data type	Sensitivity analysis	Reasoning
Myocarditis/pericarditis	Medication	Removal of NSAIDs^a^ from our list of qualifying medication supporting evidence	NSAIDs are medications that can be used to treat many different conditions besides myocarditis and pericarditis.
Myocarditis/pericarditis	Diagnostic code	Stratification by diagnostic code (myocarditis vs pericarditis)	Diagnostic criteria differ for these related conditions and may lead to different performance.
GBS^b^	Medication	Removal of gabapentin from our list of qualifying medication supporting evidence	Gabapentin was originally used as supporting evidence of a GBS episode due to its use for nerve pain associated with GBS events [44]. However, it is also used for a variety of other conditions with neuropathic pain and is not specific to GBS.
GBS	Case definition	Update case definition criteria to allow for a case to be validated as positive if there is a missing documentation for absent or diminished reflexes in the weak limbs, CSF^c^ WBC^d^ count with neurology consult, or clinical note indicating evidence of the test result of GBS more generally	Documentation required for definite or probable GBS as defined by the case definition diagnosis was often missing from our data set due to failure to capture in EHR^e^ or failure to translate to our data set and can be supplemented by an expert’s judgment (eg, a neurologist).
Febrile seizure	Medication	Addition of medications used to treat fever	The original febrile seizure algorithm did not filter out cases without suggested evidence, but we believed adding suggested evidence could improve PPV^f^.
Febrile seizure	Observation	Addition of observation of clinician describing the symptoms of seizure activity	The original febrile seizure algorithm did not filter out cases without suggested evidence, but we believed adding suggested evidence could improve PPV.
TTS^g^	Diagnostic code	Stratification by most prevalent diagnostic code I81 versus all other codes	Diagnostic criteria differ for these related conditions and may lead to different performance.

^a^NSAID: nonsteroidal anti-inflammatory drug.

^b^GBS: Guillain-Barré syndrome.

^c^CSF: cerebrospinal fluid.

^d^WBC: white blood cell.

^e^EHR: electronic health record.

^f^PPV: positive predictive value.

^g^TTS: thrombosis with thrombocytopenia syndrome.

Second, case definition requirements for GBS are extremely strict, and physicians in this study believed that some of these might have represented valid GBS cases while not meeting every requirement. For example, several of the cases with missing CSF WBC count did mention cytoalbuminologic dissociation (or similar); in the presence of such a clinical statement, we might infer that CSF WBC count was performed and acceptable to meet the case definition criteria despite a missing test result.

Furthermore, in cases where a neurologist felt strongly that GBS was a likely diagnosis, along with other supporting evidence, it may be acceptable to rely on documented progressive and significant muscle weakness, especially with conflicting reflex findings. In these instances, we placed more weight on the clinician review (which may account for any unforeseen difficulties in data processing and the strictness of the case definition), not relying solely on the available (nonmissing) data types of the algorithm for assigning case diagnostic certainty.

## Results

### Population Sample

Figure 2 illustrates the identification of the study populations and validation sample. From the study population of 20.7 million medical encounters for 2,666,974 patients over the study period, the algorithm selected 1195 (0.04%) cases of myocarditis/pericarditis, 550 (0.02%) of anaphylaxis, 123 (0.005%) of GBS, 626 (0.02%) of febrile seizure, and 395 (0.01%) of TTS. Of these patient cases (n=2,666,974), a stratified, random sample of 135 (0.01%) cases each was selected from myocarditis/pericarditis, 135 (0.01%) from anaphylaxis, and 135 (0.01%) from TTS populations. All 75 pre-EUA cases of GBS and a random sample of 35 post-EUA cases were selected to be validated. A random selection of 100 cases from the pre-EUA period were sampled to validate febrile seizure. An additional 27 negative control cases were sampled for each algorithm from the roughly 20.7 million medical encounters not selected by the algorithm in our study period. In total, 20 of these cases were sampled from the period before the COVID-19 vaccine EUA, and the remaining 7 came from the period after the EUA.

### Overall PPV and Interrater Reliability Results

Table 4 presents algorithm performance measured by PPV for each of the 5 AESIs using cases that had sufficient evidence and all cases (ie, including cases unable to be confirmed as positive by clinicians due to insufficient evidence). Counts for the number of cases included in each PPV calculation can be found in Table S3 in Multimedia Appendix 1 [21-23,25-27].

Overall PPVs, when removing all cases with insufficient evidence, were highest for anaphylaxis (93.3%, 95% CI 86.4%-97%) and febrile seizure (89%, 95% CI 80%-94.4%), followed by myocarditis/pericarditis (83.5%, 95% CI 74.9%-89.6%) and TTS at unusual sites (70.2%, 95% CI 61.4%-77.6%). The lowest was for GBS (47.2%, 95% CI 35.8%-58.9%). All negative control cases across the 5 phenotypes were correctly classified by the algorithms.

The PPV results from the chart reviews of the validation sample for each AESI are reported for all cases as well as for only cases with sufficient evidence to make a clear by chart reviewers. The frequencies and percentages for insufficient evidence are presented with the stratification results in Table 5. The interrater reliability scores for clinician chart reviews all showed substantial agreement between the clinicians (Table 6). Interrater reliability, measured by Cohen κ, suggests substantial reliability when the value is >0.61, with many similar texts recommending a higher threshold of 0.80 [43].

**Table 4:** Total validation positive predictive value (PPV) results.

AESI^a^ and metric			Detected cases, PPV % (95% CI)
**Myocarditis/pericarditis**			
	Cases with sufficient evidence only	83.5 (74.9-89.6)	
	All cases	63.7 (55.2-71.4)	
**Anaphylaxis**			
	Cases with sufficient evidence only	93.3 (86.4-97)	
	All cases	72.6 (64.4-79.5)	
**GBS^b^**			
	Cases with sufficient evidence only	47.2 (35.8-58.9)	
	All cases	30.9 (22.9-40.3)	
**TTS^c^**			
	Cases with sufficient evidence only	70.2 (61.4-77.6)	
	All cases	64.4 (55.9-72.1)	
**Febrile seizure**			
	Cases with sufficient evidence only	89 (80-94.4)	
	All cases	89 (80-94.4)	

^a^AESI: adverse event of special interest.

^b^GBS: Guillain-Barré syndrome.

^c^TTS: thrombosis with thrombocytopenia syndrome.

**Table 5:** Stratification analysis: validation sample results.

AESI^a^ and metric			Detected cases		Pre-EUA^b^ period		Post-EUA period		Inpatient		Outpatient		Emergency department
**Myocarditis/pericarditis (n=135)**													
	Total cases, n	135		100		35		91		26		18	
	Total TP^c^ cases, n (PPV %; 95% CI)	86 (63.7; 55.2-71.4)		68 (68.0; 58.1-76.5)		18 (51; 35-68)		72 (79; 69-86)		10 (38; 21-59)		4 (22; 7-48)	
	Total cases with sufficient evidence, n (PPV^d^ % for TP cases with sufficient evidence; 95% CI)	103 (83.5; 74.9-89.6)		79 (86; 76-92)		24 (75; 53-89)		79 (91; 82-96)		16 (63; 36-84)		8 (50; 15-85)	
**Anaphylaxis (n=135)**													
	Total cases, n	135		100		35		27		—^e^		108	
	Total TP cases, n (PPV %; 95% CI)	98 (72.6; 64.4-79.5)		70 (70; 60.2-78.3)		28 (80; 63-90.9)		17 (63; 42.9-79.7)		—		81 (75; 65.8-82.4)	
	Total cases with sufficient evidence, n (PPV %; 95% CI)	105 (93.3; 86.4-97)		74 (94.6; 86.2-98.4)		31 (90.3; 73.4-98)		19 (89.5; 65.6-99.7)		—		86 (94.2; 86.6-97.9)	
**GBS^f^ (n=110)**													
	Total cases, n (%)	110		65		45		110		—		—	
	Total TP cases, n (PPV %; 95% CI)	34 (30.9; 22.9-40.3)		24 (40; 25.9-49.5)		20 (44; 30.4-59.4)		34 (30.9; 22.8-40.3)		—		—	
	Total cases with sufficient evidence, n (PPV %; 95% CI)	72 (47.2; 35.8-58.9)		52 (46.2; 32.9-60)		20 (50; 28.1-71.9)		72 (47.2; 35.8-58.9)		—		—	
**TTS^g^ (n=135)**													
	Total cases, n	135		100		35		133		1		1	
	Total TP cases, n (PPV %; 95% CI)	87 (64.4; 55.9-72.1)		64 (64; 54-72.9)		23 (66; 48.2- 80)		86 (64.7; 56.1-72.4)		1 (100; 0-100)		0 (0; 0-100)	
	Total cases with sufficient evidence, n (PPV %; 95% CI)	124 (70.2; 61.4-77.6)		91 (70.3; 60-78.9)		33 (70; 51.6-83.5)		122 (70.5; 61.7-78)		1 (100; 0-100)		1 (100; 0-100)	
**Febrile seizure (n=100)**													
	Total cases, n	100		100		—		1		—		99	
	Total TP cases, n (PPV %; 95% CI)	73 (73; 63.3-80.9)		73; (73; 63.3-80.9)		—		0 (0; 0-100)		—		73 (74; 64.1-81.6)	
	Total cases with sufficient evidence, n (PPV %; 95% CI)	83 (88; 78.8-93.6)		83 (88; 78.8-93.6)		—		1 (0; 0-100)		—		82 (89; 80-94.4)	

^a^AESI: adverse event of special interest.

^b^EUA: emergency use authorization.

^c^TP: true positive.

^d^PPV: positive predictive value.

^e^Not applicable.

^f^GBS: Guillain-Barré syndrome.

^g^TTS: thrombosis with thrombocytopenia syndrome.

**Table 6:** Interrater reliability.

AESI^a^	Total cases validated, n	Interrater reliability
Myocarditis/pericarditis	162	0.814
Anaphylaxis	162	0.770
GBS^b^	137	0.832
TTS^c^ at unusual sites	162	0.851
Febrile seizure	120	0.965

^a^AESI: adverse event of special interest.

^b^GBS: Guillain-Barré syndrome.

^c^TTS: thrombosis with thrombocytopenia syndrome.

### Stratification

To evaluate consistency across pre- and post-EUA periods and care settings, we reported true positive and PPV results for each stratum (Table 5).

None of the algorithms had notable differences between the pre- and post-EUA periods since all 95% CIs had some overlap. However, there were some differences between the PPVs for the 2 periods that could be significant with a larger validation sample. The difference in PPV for myocarditis/pericarditis varied from 68% in the pre-EUA period to 51.4% in the post-EUA period, while anaphylaxis showed the opposite pattern with a 70% PPV in the pre-EUA period that increased to 80% PPV in the post-EUA period.

We also reported stratified results by care setting (Table 5). For myocarditis/pericarditis, the PPV of cases with an inpatient care setting (79.1%, 95% CI 69.4%-86.4%) was notably higher than that from the outpatient (38.5%, 95% CI 21.2%-58.8%) or emergency department (22.2%, 95% CI 6.7%-47.9%) care settings.

Anaphylaxis did not have a large difference across care settings, as the 95% CIs overlapped between the 2 care settings. However, they did show better performance with cases in an emergency department (PPV 75%, 95% CI 65.8%-82.4%) care setting over cases with an inpatient care setting (PPV 63%, 95% CI 42.9%-79.7%). The other AESI algorithms filtered for only 1 care setting or had a vast majority of cases in 1 care setting.

### Sensitivity Analysis

#### Medication and Observation Algorithm Changes

We analyzed whether changes to medication code lists for the myocarditis/pericarditis and GBS algorithms could improve performance. For the myocarditis/pericarditis algorithm, removal of nonsteroidal anti-inflammatory drugs from the medication code lists showed no change in PPV at 83.5% (Table 7), but PPV values were higher for cases selected with the pericarditis instead of myocarditis *ICD-10* codes.

For the GBS algorithm, when cases were removed where gabapentin (used for post-GBS pain management) was the only supporting evidence, PPV increased to 38.1% (95% CI 28.2%-49.1%) from 30.9% (95% CI 22.9%-40.3%; Table 8).

Our initial febrile seizure algorithm did not use any supporting evidence to filter out possible false positives since we believed we could get adequate PPV without it.

For our sensitivity analysis, we tested requiring supporting evidence in the condition period, such as the presence of medications for reducing fever such as acetaminophen, observation evidence when the patient’s chief complaint was related to fever or seizure, or the presence of both. When filtered to only cases with either medication or observation evidence, febrile seizure PPV increased significantly to 93.3% (95% CI 84.7%-97.6%) from the original algorithm PPV of 73% (95% CI 63.3%-80.9%), with no overlap in 95% CIs and a *P* value of <.001 (Table 9). When the algorithm required both medication and observation evidence, it performed even better (PPV 96.9%, 95% CI 88.5%-99.9%).

**Table 7:** Sensitivity analysis: myocarditis/pericarditis validation sample results.

AESI^a^ and sensitivity analysis	Total TP^b^ cases, n	Selected cases, n (change, n)^c^	PPV^c^, % (95% CI; change)^d^	Selected cases with sufficient evidence, n (change, n)^d^	PPV, % (95% CI; change)
Removal of NSAIDs^e^	86	135 (0)	63.7 (55.2-71.4; 0)	103 (0)	83.5 (74.9-89.6; 0)
Pericarditis diagnosis^f^	59	82 (–53)	72 (61.1-80.8; +8.3)	67 (–36)	88.1 (77.6-94.3; +4.6)
Myocarditis diagnosis^f^	27	53 (–82)	50.9 (37.4-64.3; –12.8)	36 (–67)	75 (57.9- 87.1; –8.5)

^a^AESI: adverse event of special interest.

^b^TP: true positive.

^c^PPV: positive predictive value.

^d^Values in parentheses reflect the change due to the modified algorithm features.

^e^NASID: nonsteroidal anti-inflammatory drug.

^f^All International Classification of Diseases, Tenth Revision, Clinical Diagnosis codes that the algorithm used were broken into 2 groups: myocarditis (I40.0 infective myocarditis, I40.1 isolated myocarditis, I40.8 other acute myocarditis, I40.9 acute myocarditis, unspecified, and I51.4 Viral myocarditis) and pericarditis (B33.22 viral pericarditis, B33.23 acute rheumatic pericarditis, I30.0 acute nonspecific idiopathic pericarditis, I30.1 infective pericarditis, I30.8 other forms of acute pericarditis, I30.9 acute pericarditis, unspecified, I32 pericarditis in diseases classified elsewhere, and I41 meningococcal pericarditis).

**Table 8:** Sensitivity analysis: Guillain-Barré syndrome validation sample results.

AESI^a^ and sensitivity analysis	Total TP^b^ cases	Selected cases (change)^c^	PPV^c^, % (95% CI; change)^d^	Selected cases with sufficient evidence (change)^d^	PPV, % (95% CI; change)^d^
Removal of gabapentin	33	86 (–24)	38.4 (28.6-49.2; 7.5)	53 (–19)	62.3 (48.3-74.5; +15)
Adjusted case definition	49	110 (0)	44.5 (35.4-54; +13.6)	72 (0)	68.1 (56.3-78; +20.8)
Adjusted case definition+removal of gabapentin	49	86 (–26)	57.1 (46.2-67.4; +26.2)	68 (–4)	72.1 (60-81.6; +24.8)

^a^AESI: adverse event of special interest.

^b^TP: true positive.

^c^PPV: positive predictive value.

^d^Values in parentheses reflect the change due to the modified algorithm features.

**Table 9:** Sensitivity analysis: febrile seizure.

AESI^a^ and sensitivity analysis	Total TP^b^ cases, n	Selected cases, n (change, n)^c^	PPV^d^, % (95% CI; change)^c^	Selected cases with sufficient evidence, n (change, n)^c^	PPV^d^, % (95% CI; change)^c^
Cases with either medication or observation	70	75 (–25)	93.3 (84.7-97.6; +20.3)	73 (–10)	95.9 (87.9-99.2; +7.9)
Cases with both medication and observation evidence	63	65 (–35)	96.9 (88.5-99.9; +23.9)	63 (–20)	100 (92.8-100; +12)

^a^AESI: adverse event of special interest.

^b^TP: true positive.

^c^Values in parentheses reflect the change due to the modified algorithm features.

^d^PPV: positive predictive value.

#### Diagnostic Code List Changes

We also analyzed if changing diagnostic codes that were used to identify the AESI might lead to higher performance for the myocarditis/pericarditis and TTS algorithms.

For myocarditis/pericarditis, we found that an algorithm only looking for the myocarditis code (PPV 50.9%, 95% CI 37.4%-64.3%) underperformed an algorithm with just pericarditis codes (PPV 72%, 95% CI 61.1%-80.8%; Table 7). For TTS, we found that the main *ICD-10* code I81 for “portal vein thrombosis” (73.5%, 95% CI 64%-81.3%) outperformed all other codes in our code list, including G08 (intracranial and intraspinal phlebitis and thrombophlebitis), I82.0 (Budd-Chiari syndrome), I82.3 (embolism and thrombosis of renal vein), and I82.890 (acute embolism and thrombosis of other specified veins), with a PPV of 36.4% (95% CI 21.3%-54.4%; Table 10).

**Table 10:** Sensitivity analysis: thrombosis with thrombocytopenia syndrome (TTS).

AESI^a^ and sensitivity analysis	Total TP^b^ cases, n	Selected cases, n (change, n)^c^	PPV^d^, % (95% CI; change)^c^	Selected cases with sufficient evidence, n (change, n)^c^	PPV, % (95% CI; change)^c^
I81	75	102 (–33)	73.5 (64-81.3; +9.1)	96 (–28)	78.1 (68.6-85.4; +8)
All other TTS *ICD*^e^ codes^f^	12	33 (–102)	36.4 (21.3-54.4; –28)	28 (–96)	42.9 (25.4-62.1; –27.3)

^a^AESI: adverse event of special interest.

^b^TP: true positive.

^c^Values in parentheses reflect the change due to the modified algorithm features.

^d^PPV: positive predictive value.

^e^ICD: International Classification of Diseases.

^f^All other TTS *ICD* codes include G08, I82.0, I82.3, and I82.890.

#### Case Definition Validation Criteria

Finally, we analyzed whether a small update to our case definition criteria for the GBS algorithms described in the Stratification Analysis and Sensitivity Analysis section would improve reported performance in Table 7. When we applied both changes, the validation criteria change to the algorithm and removal of gabapentin, as discussed in the Medication and Observation Algorithm Changes section, the algorithm achieved a PPV of 57.1% (95% CI 46.2%-67.4%).

## Discussion

### Principal Findings

#### Overview

The results of this study show that for 4 out of 5 AESIs, we can build an interoperable computable phenotype with comparable or increased performance to algorithms in the existing literature. These algorithms are developed using a rules-based approach to facilitate their application and increase the generalizability of performance across EHR databases. For the phenotypes with poorer performance, the issues were often that the case definition required documentation of a test that was lost in our data pipeline, or was not completed, or was not recorded by the treating physician or nurse. While these cases are marked as false positives based on our methodology, they may be true AEs that are lacking the documentation to meet the case definition. Some small updates to the algorithms or the case definition evaluation method could be made to potentially improve the algorithms’ performances, but a more important next step would be to validate our algorithms on other data partners to ensure generalizability of the original algorithms and any updates. Given the need for active AE surveillance, this study is still an important first step toward building an algorithm that can be distributed and implemented on health provider EHR databases and can accurately detect AEs.

The PPV results of the phenotypes, negative control groups, and stratification and sensitivity analysis are discussed in more detail in the following sections. Note our negative control groups and many of the stratification and sensitivity analyses have sample sizes too small to draw strong conclusions as illustrated by the width of the 95% CIs for those results. These were exploratory analyses completed as a supplement to the main findings of the study around the PPV of the algorithms.

#### Myocarditis/Pericarditis

The myocarditis/pericarditis algorithm showed strong PPV performance using cases with sufficient evidence. The literature appears to lack good comparison studies against which to evaluate this algorithm’s performance. A meta-analysis from 2013 reviewed myocarditis/pericarditis algorithm studies and found that none of them evaluated their algorithm by calculating PPV [45].

When myocarditis/pericarditis was segmented via care settings, algorithm performance was highest for inpatient settings, with a PPV of 79.1%. This can be attributed to the availability of supporting clinical data needed for accurate case detection in such settings. Given that inpatient testing is necessary to meet the criteria of the case definition, the algorithm performance matches clinical expectations and adds to its public health importance.

In emergency care settings, myocarditis/pericarditis is often diagnosed for patients with a history of inpatient visits to one or more other health systems. This increases the probability of these patients having additional documentation necessary to meet the case definition. This highlights the role of health information exchanges in supporting public health use cases, improving AE reporting, and enhancing postmarket surveillance.

Myocarditis/pericarditis had a notable difference in PPVs for pre- and post-EUA date. The post-EUA date strata of the sample had a higher percentage of cases coming from the emergency department, which had few cases before EUA. This could be explained by patients being diagnosed during previous inpatient stays in other health systems and a lower threshold to provide a preliminary diagnosis with limited information. This category had a lower PPV on average for myocarditis/pericarditis, likely due to less documentation in an emergency care setting than in an inpatient care setting. This highlights the need for further validation of the algorithm in these settings for an effective public health benefit and to gain confidence that our algorithm is fit for purpose. Because the aim of the algorithms is postvaccination AESI detection in support of public health safety surveillance, any potential degradation in performance in the post-EUA period is a concern. If performance decrease in the post-EUA period is driven by postvaccination myocarditis/pericarditis being more likely to have confounding physical findings that could affect how quickly and in which care setting it gets diagnosed, the PPV from this study may not be applicable to a postvaccination version of the phenotype. There is a small overlap in the 2 periods’ PPV 95% CI, and a 2-sample proportion test returns a *P* value of .08. This suggests that the difference could also be due to statistical noise. However, given the importance of the post-EUA period to the algorithm’s future task and the size of the difference, we suggest validating additional cases in the post-EUA period to confirm whether the algorithm is actually less effective.

#### Anaphylaxis

In cases with sufficient evidence, our anaphylaxis algorithm performed strongly with a PPV score of 93.3% (95% CI 86.4%-97%). This shows a possible slight improvement over previous anaphylaxis research, although both results were within the 95% CI [33,34]. When stratified by care setting, the algorithm performed better in emergency department care settings. This can be explained due to the anaphylaxis symptoms and treatment being more likely to be well-documented in this setting. Availability of additional evidence increases the PPV of the algorithm. Since anaphylaxis cases related to vaccination are more likely to culminate in visits to the emergency department, the better performance of the algorithm would provide a better public health benefit.

Overall, the performance of the algorithm was moderate compared with that seen in literature. With no obvious avenues for improvement available, no additional sensitivity analyses were applied.

#### GBS Algorithm

Our initial GBS algorithm showed weak performance for GBS with a PPV of 47.2% (95% CI 35.8%-58.9%). Given existing research on GBS validations, this result is not surprising, since our result is comparable with a study result showing GBS algorithm validation PPV of 29% (95% CI 24%-34%) [35]. We hoped that our algorithm would improve on this study’s results, allowing us to meet the “moderate” performance threshold defined in the Methods section, given that we added additional logic to require suggested evidence and filter out historical diagnoses. However, we believe that the algorithm’s performance could be improved based on the sensitivity analysis results.

An increase in performance was observed when adjusting the case definition interpretation of GBS to allow for more general written clinical notes or neurology consult evidence to replace specific documented test results. The lack of standardization in laboratory results is fraught with challenges such as inconsistent data. The observed improvement in the GBS phenotype highlighted the need for further standardization to have a better impact on public health benefit.

Furthermore, the performance of the GBS algorithm was improved by the exclusion of nonspecific medications such as gabapentin, increasing its public health benefit. Gabapentin is often used to treat generalized neuropathic pain for a variety of conditions other than GBS, including diabetes, and can confound the results.

With both case definition and medication adjustments to the algorithm, the PPV rose to be closer to the moderate performance threshold and an increase over the cited historical study [35]. Because these changes were informed by the cases in the validation study post hoc, they might be overfitted to this validation sample and may not be generalizable. They should be tested in other EHR systems.

The GBS algorithm performed slightly better in the post-EUA period, but the performance of both periods was well within the 95% CI of the other period. The GBS algorithm only applies to the inpatient care setting; therefore, no care setting stratification analysis was performed.

#### Febrile Seizure

Our febrile seizure algorithm performed strongly, with a PPV score of 89% using cases with sufficient evidence. This performance is in line with existing febrile seizure algorithm validation research [32], where a febrile seizure validation study on the FDA Sentinel database showed a PPV of 70% (95% CI 64%-76%). Our sensitivity analysis suggests that even better performance could possibly be achieved by adding additional filters to select cases with supporting medication and observation evidence, which are well-documented in EHRs. The better performance of the algorithm provides better public health benefits and further supports the use of EHRs in the detection of AEs. For cases that met either or both criteria, the PPV increased. Since these changes to the algorithm happened after the validation was completed, they overstate the general performance increases when applied to a new EHR setting but offer avenues for a future validation study. Future research can test whether stronger performance is possible with these filters and focus on reviewing the algorithm’s application to AEs following pediatric vaccinations.

#### TTS Algorithm

The TTS algorithm showed moderate performance for PPV at 70.2% which is similar to a separate FDA TTS validation study which estimated the performance at 76.1% (95% CI 67.2-83.2%) [29]. TTS had consistent performance across both pre- and post-EUA periods and did not have enough cases in the outpatient and emergency department care settings for any defensible findings around diagnosis care setting stratification. Our sensitivity analysis revealed that when the AESI was diagnosed with the *ICD-10* code I81 (portal vein thrombosis), the algorithm showed a significant increase when compared with the performance of all other *ICD* codes (73.5%, 95% CI 64%-81.3%, compared with 36.4%, 95% CI 21.3%-54.4%). Although if an increase to specificity is desired at the cost of some sensitivity, the TTS algorithm could be limited to only select the higher performing I81 diagnosis code.

### Limitations

There are several limitations to this study. First, it only evaluates general AESIs and not postvaccination AESIs specifically since the algorithms do not require the evidence of vaccine administration as criteria. While this was necessary due to the rareness of the postvaccination AESIs in our data, it is possible that the algorithms perform worse detecting postvaccination AESIs specifically since they will often present slightly differently in different populations when occurring after a vaccine administration. For example, the major presenting symptoms appeared to resolve faster in cases of myocarditis after COVID-19 vaccination than in typical viral cases of myocarditis [9]. To guard against this, we included both pre– and post–COVID-19 EUA data with the hope that post-EUA cases would include some postvaccination AESIs. However, we did not have enough post-EUA cases available to build a large enough sample size for a comparison with sufficient statistical power to provide definitive evidence on this topic. Another limitation in this vein is the general small sample size for all stratification, sensitivity, and negative control analyses. We make sure to state that these analyses are exploratory in nature, and the reader should not form strong conclusions from them given their small samples size and large CI range. Future research could address these concerns by identifying a data source with enough postvaccination AESI cases to complete a comparably large validation study.

An additional limitation of this study is that it only measures algorithms’ PPVs instead of investigating other metrics that could give a better picture of the algorithm’s holistic performance such as sensitivity and specificity. Specifically, these other metrics would estimate how many of the total positive cases are being identified and how well the algorithm is able to identify cases without the AESIs. However, we believe that this limitation is necessary for the following reasons: (1) the main purpose of this study was to assess the PPV of phenotypes because it answers the most relevant public health question, if the algorithms will generate a quality detected set of AE cases for the public health surveillance and (2) a much higher cost and more extensive data sharing are needed to properly estimate sensitivity and specificity because of the required validation sample size necessary for a negative control group. To calculate PPV, one only needs a sample of the cases selected by the algorithm. To estimate the sensitivity and specificity, however, it would be necessary to also validate an extremely large negative control group sample since the AESI conditions that the algorithms try to detect are often rare events. We would expect it to be even more rare for these conditions of interest for AESIs to happen and not be recorded with types of structured data elements that are being used in the phenotypes. In fact, the lack of structured data elements in some negative control cases led to a clinician asking the research team if something was wrong because their case had no relevant charted events to be reviewed. A much larger validation study would also expose clinicians to a larger set of patient data for cases that have a low likelihood of having an AE. This approach limits the interaction with protected health information data until the algorithms’ PPVs support continued research with broader samples and methodologies.

Another limitation is that although they were designed to be simple to deploy, the algorithms are still time-consuming to apply to different EHR systems. Although a hallmark of this algorithm is its interoperability, the algorithm logic still must be applied to the EHR common data model or extracted and translated into another common data model as was done for this study. Interoperable codes should be available for all patients, given the requirement to provide patient data in an interoperable FHIR standard. However, given the recency of the requirement, they might not be available in all systems and require some code translation on the health organization side, especially when analyzing at the population level. In addition, since the interoperable codes will only be available through a FHIR API, this adds another data pull and integration with the EHR system to obtain these codes for the algorithm.

In the future, the evolving landscape of health IT may facilitate the public health use cases of detecting and reporting postvaccination AESIs in a safe and secure manner that protects patient privacy. This could be achieved by EHRs supporting secure querying of patient cohorts with probable postvaccination AESIs using clinical query language [46] or other interoperable query language. Reducing the burden of automatic detection of postvaccination AESIs would help public health organizations improve AE surveillance with minimal additional burden to health care organizations and providers.

A final limitation of this study is that the algorithms were only applied to 1 site. Going forward, algorithm performance should be validated at other sites to ensure their generalizability. Although the algorithms were generated without prior input from the data, the study is still limited to 1 health care organization, and this method could have different operating characteristics (PPV, sensitivity, etc) at a second location.

Future research can be performed to improve algorithm accuracy and as stated previously would require additional partner EHR data systems. To create a better performing algorithm, machine learning techniques could be used to train the model to identify specific patterns of data instead of relying on rules-based methods that incorporate published case definition criteria and clinical subject matter expert experience. When given enough data, machine learning approaches generally outperform rules-based approaches across domains, and some prior research suggests that this is true in the medical domain as well [47].

However, machine learning methods will not generalize across EHR systems because the data patterns that machine learning identifies could be specific to an individual health care organization. Trying to build a large data set that combines multisite data is extremely difficult and costly due to concerns over infrastructure, regulations, privacy, and data standardization. A method such as federated learning could be explored to alleviate this problem. Federated learning allows multiple sites to collaboratively train a global model without directly sharing data and has been used to train machine learning algorithms at EHR sites previously [48].

### Conclusions

In summary, this study presents strong initial evidence that creating simple, interoperable, rules-based phenotypes can detect AESIs on a new data source and that the phenotypes outperform the PPV outcomes for historical validations studies for these conditions. The study validates 5 different AESIs to prove that this approach can work for a broad range of AESIs, while also highlighting where the approach might be less successful. For example, the GBS algorithm was built using *ICD-10* codes that previous validation studies have demonstrated are not accurate predictors of a GBS case that meets case definition criteria; subsequently, our GBS algorithm performed poorly. The validation study sample sizes for all AESIs allowed for adequate precision to evaluate algorithm PPV against historical studies.

An active surveillance system can enhance vaccine safety and aid in the development and use of safer vaccines and recommendations to minimize the AE risks after vaccination [49]. The algorithms were developed using a method that should be able to be applied to and generalize performance for new EHR databases, but more research is needed to confirm this. If the methodology can be successfully used to detect postvaccination AESI cases across EHR databases, these algorithms could be deployed widely to inform FDA decision-making, promote public safety, and improve public confidence. Going forward, further research and investigation are needed to enhance algorithm performance and integrate the algorithms across health care organizations for active surveillance in the interest of public health.

## Appendix

Multimedia Appendix 1 Search terms and code lists for 5 developed phenotypes and detailed case definitions.

## Abbreviations

AE: adverse event
AESI: adverse event of special interest
API: application programming interface
BEST: Biologics Effectiveness and Safety Initiative
CBER: Center for Biologics Evaluation and Research
CDC: Centers for Disease Control and Prevention
CSF: cerebrospinal fluid
EHR: electronic health record
EUA: emergency use authorization
FDA: Food and Drug Administration
FHIR: Fast Healthcare Interoperable Resources
GBS: Guillain-Barré syndrome
HAPI: Health Level 7 application programming interface
ICD-10: International Classification of Diseases, 10th Revision, clinical modification
OHDSI: Observational Health Data Sciences and Informatics
PPV: positive predictive value
RWD: real-world data
TTS: thrombosis with thrombocytopenia syndrome
USCDI: United States Core Data for Interoperability
VAERS: Vaccine Adverse Event Reporting System
WBC: white blood cell

## Data Availability

The data sets generated during and analyzed during this study are not publicly available, and were only made available to the Food and Drug Administration for the purpose of evaluating algorithms for adverse events of special interest outcomes. For inquiries or questions regarding the data, individual queries should be directed to the corresponding author.
