# Supplementary material for: Development of Interoperable Computable Phenotype Algorithms for Adverse Events of Special Interest to Be Used for Biologics Safety Surveillance: Validation Study
Source: JMIR Public Health Surveill. 2024 Jul 15;10:e49811. doi: 10.2196/49811 (PMC11287092; doi:10.2196/49811)
Supplement: Multimedia Appendix 1 [file publichealth_v10i1e49811_app1.docx]

**Development of Interoperable Computable Phenotype Algorithms for Adverse Events of Special Interest to Be Used for Biologics Safety Surveillance: Validation Study**

*Multimedia Appendix*

**Document Description**

This is a Multimedia Appendix to a full manuscript published in the J Med Internet Res. For full copyright and citation information see <http://dx.doi.org/10.2196/49811>.

Table S1 AESI Search Term and codelists Appendix

| **AESI** | **Data Element** | **Concept** | **Search Terms** |
| --- | --- | --- | --- |
| Myocarditis / pericarditis | Labs & Observations | Creatine kinase | creatine, kinase |
|  |  | Ejection Fraction | ejection, fraction |
|  |  | Myocardial band | sedimentation, reactive, protein, complete, blood, count |
|  |  | Troponin | troponin |
|  | Medication | ACE Inhibitors | lisinopril, benazepril, enalapril, ramipril, quinapril, fosinopril, captopril, peridopril, trandolapril, moexipril, imidapril, enalaprilat, cilazapril, zofenopril, spirapril, delapril hydrochloride, temocapril hydrochloride |
|  |  | Anti-Inflammatory | NDC list |
|  |  | ARBs | losartan, valsartan, olmesartan, irbesartan, talmisartan, candesartan, azilsartan, eprosartan |
|  |  | Beta-adrenergic blockers | metoprolol, atenolol, carvedilol, popanolol, timolol, bisoprolol, nebivolol, labetalol, sotalol, nadolol, acebutolol, betaxolol, esmolol, pindolol, carteolol, celiprolol, oxprenolol, penbutolol, talinolol, tertatolol, alprenolol, bopindolol, bupranolol, mepindolol, practolol |
|  |  | Phosphodiesterase inhibitors | milrinone, inamrinone, enoximone, buclasdine sodium |
|  |  | Vasodilators | isosorbide, nitrolycerin, isosorbide dinitrate, nicorandil, heptaminol, molsidomine, pentaerythritol, nesiritide, trapidil, erythryityl tetranitrate, flosequinan, oxyfedrine, hexobendine, prenylamine, efloxate, chromonar |
|  | Procedure | Electrocardiogram | Electrocardiogram |
| Anaphylaxis | Medication | Antihistamine H1 | diphenhydramine, hydroxyzine, dimetindene, doxylamine, promethazine, clemastine, dimenhydrinate |
|  |  | Antihistamine H2 | ranitidine, famotidine |
|  |  | Steroids | prednisone, cortisone, predisolone, dexamethasone |
|  |  | Sympathomimetic | epinepherine, ephedrine, racepinepherine |
|  | Procedure | Intubation | 45887795, 42738853, 42738852, 2788038, 2788037, 2788036, 2788027, 2788026, 2788025, 2788024, 2788022, 2788021, 2788020, 2788019, 2788017, 2788016, 2787824, 2787823, 2745447, 2745444, 2745440, 2514578, 2314036, 2314035, 2314003, 2314002, 2314001, 2314000, 2108681, 2106642, 2106470, 2106469 |
| GBS | Labs & Observations | CSF | 2879-5', '55787-6' |
|  | Medication | Pain Relief / Anticonvulsant | gabapentin, carbamazepine |
|  |  | IVIg |  |
| Febrile Seizure | Condition (Exclusion Criteria) | Central Nervous System Infection |  |
|  |  | Epilepsy |  |
| TTS | Labs & Observations | Platelet Count | '776-5', '26515-7', '777-3', '49497-1', '778-1', '74464-9', '26516-5', '13056-7', '74775-8' |
|  |  | Doppler Ultrasound | Doppler ultrasound, duplex |
|  |  | CT Angiography | Computed tomography angiography, CT angiography, CT angiography impression |
|  |  | Conventional angiography | Conventional angiography, angiography impression, angiography |
|  |  | Digital subtraction angiography | Digital subtraction angiography, DSA |
|  |  | thrombectomy | thrombectomy |
|  |  | D-Dimer | D-Dimer |
|  |  | PF4 | PF4, Positive anti-platelet factor 4, positive anti platelet factor 4 |
|  | Procedure | thrombectomy | thrombectomy |
|  |  | Radiology | 40757120, 40757093, 40757042, 40756941, 40756939, 40756863, 40756829, 2793856, 2793855, 2793854, 2793853, 2793852, 2793851, 2793850, 2793849, 2793848, 2793847, 2793846, 2793562, 2793561, 2793560, 2793559, 2793558, 2793557, 2793556, 2793555, 2793542, 2793541, 2793540, 2793539, 2793538, 2793537, 2793536, 2793535, 2793534, 2793333, 2792855, 2792854, 2792853, 2792852, 2792851, 2792850, 2792849, 2792848, 2792847, 2792846, 2792845, 2792844, 2792843, 2792842, 2791261, 2791260, 2791259, 2791258, 2787681, 2787680, 2787679, 2787678, 2787677, 2787676, 2787675, 2787344, 2787343, 2787342, 2787341, 2787340, 2787339, 2787338, 2787337, 2787336, 2787335, 2787119, 2787118, 2787117, 2787116, 2787115, 2786161, 2786160, 2786159, 2786158, 2786157, 2786156, 2786155, 2786154, 2786153, 2786152, 2786151, 2786150, 2786149, 2786148, 2786147, 2786146, 2786145, 2786144, 2786143, 2786142, 2786141, 2786140, 2786139, 2786138, 2785893, 2785892, 2785891, 2785890, 2785889, 2785888, 2785887, 2785886, 2785837, 2785836, 2785835, 2785834, 2785833, 2785832, 2785831, 2785830, 2785829, 2785828, 2785827, 2785826, 2785825, 2785824, 2785823, 2785822, 2785821, 2785820, 2785819, 2785818, 2785817, 2785816, 2785815, 2785814, 2211406, 2211405, 2211404, 2211381, 2211380, 2211379, 2211378, 2006697, 2211382, 2211385, 2211384, 2211383, 45890131, 2211382, 2211385, 2211384, 2616254, 2616256, 2616255, 2211383, 2827634, 2807682, 2807683, 2827635, 2793094, 2793093, 36911821, 36908692, 2006957, 2881371, 2894386, 2832036, 2897669, 2801493, 2818908, 2786399, 2786398, 2786397,  2212056, 2212056, 2106989, 2212053, 2212053, 2106971, 2793912, 2793911, 2313991, 2313990, 2313989, 2211616, 2108056, 2211617, 2211617, 2006913, 2786633, 4194532, 4194532, 2786636, 2786615, 2786622, 2786618, 2789640, 2789379, 4244397, 4244397, 2789371, 2789632, 2789631, 2789635, 2789639, 2789387, 2786619, 2786616, 2786640, 2789370, 2789378, 2789643, 2786617, 2786637, 2789642, 2786634, 2786620, 2789648, 2789374, 2789381, 2788668, 2789633, 2786621, 2789644, 2789372, 2786425, 2789382, 2789383, 2788887, 2789373, 2789641, 2789634, 2789386, 2789389, 2788888, 2788669, 2788890, 2789380, 2788666, 2788884, 2789647, 2786635, 2786639, 2788864, 2789390, 2788665, 2789636, 2789375, 2788885, 2788863, 2789629, 2786638, 2789650, 2788891, 2786632, 2789652, 2789638, 2789391, 2789377, 2789388, 2789651, 2789368, 2786380, 2789369, 2793617, 2789649, 2789385, 2788664, 2788886, 2789384, 2786378, 2786381, 2789376, 2788670, 2793612, 2786396, 2786395, 2789630, 2789637, 2786375, 2786374, 2793614, 2788883, 2788667, 2786390, 2793620, 2789646, 35622900, 4101610, 2789645, 2793618, 2786377, 2786394, 37109424, 2793615, 2793611, 2793619, 2786379, 2786389, 2786373, 2786392, 2788889, 42535657, 2786391, 37109187, 42535033, 2793616, 2786388, 42535661, 2786376, 2793613, 42535555, 42535658, 40489371, 42535034, 42535659, 2793622, 35622899, 2786393, 2793621, 37109423) |
|  |  | MRA | Magnetic resonance venography, Magnetic resonance arteriography, Magnetic resonance angiography ,MRA, MRV, 2211345, 2211350, 2211348, 2211517, 2211349, 2211504, 2211347, 2211385, 2211429, 2211346, 2211471, 2211419, 2211342 |
|  |  | Doppler Ultrasound | Doppler ultrasound, duplex |
|  |  | CT Angiography | Computed tomography angiography, CT angiography, CT angiography impression |
|  |  | Conventional angiography | Conventional angiography, angiography impression, angiography |
|  |  | Digital subtraction angiography | Digital subtraction angiography, DSA |

**Table S2:** Adverse event of special interest detailed case definitions and descriptions.

| **AESI** | **Description** | **Case Definition Reference** |
| --- | --- | --- |
| **Myocarditis and Pericarditis** | Myocarditis and pericarditis are a spectrum of illnesses and frequently occur in combination. If symptoms of both exist, the level of certainty determination may differ for each diagnosis. Clinicians chose the higher of the two levels of certainty to make a diagnosis.  Findings in the following categories determine acute myocarditis and pericarditis:   - Signs and symptoms for myocarditis include dyspnea, palpitations, and chest pain of probable cardiac origin. For pericarditis, they include typical chest pain (worsened by lying down and relieved by sitting up or leaning forward), pleuritic chest pain, or peri-cardinal rub. - Histopathology evidence of myocardial or pericardial inflammation is required, along with positive signs/symptoms for all definite determinations. For probable or possible cases, histopathology evidence isn't required. Definite determinations in the presence of positive histopathology and signs/symptoms do not require further supporting EKG, imaging, or cardiac enzyme evidence. - Cardiac enzymes are evaluated only for myocarditis determinations. For probable cases, they must exhibit elevated troponin I or T or creatine kinase–myocardial band. Definite and possible cases do not require troponin evidence. - Electrocardiogram is helpful for distinguishing possible myocarditis cases, requiring ST-segment or T-wave abnormalities, arrhythmias, or AV nodal delays. Definite or probable myocarditis determinations do not require EKG changes. For pericarditis, only probable cases require EKG changes, including diffuse ST-segment elevations or PR depressions without reciprocal ST depressions. - Imaging studies: For myocarditis probable determinations, imaging should show evidence of depressed LV function, new or increased, or myocardial inflammation. For pericarditis probable determinations, echocardiogram should show an abnormal collection of pericardial fluid. | Morgan 2008 |
| **Anaphylaxis** | Anaphylaxis hypersensitivity reactions exhibit multi-organ-system involvement and can rapidly become life-threatening. The Brighton case definition classes these symptoms into several different clinical feature (criterion) types:   - Major dermatologic or mucosal features include generalized urticaria (hives), erythema, angioedema, or pruritis with rash. - Major respiratory features include bilateral wheeze (bronchospasm), stridor (upper airway swelling of lip, tongue, throat), or respiratory distress (two signs of tachypnea, including using accessory muscles, recession, cyanosis, or grunting). - Major cardiovascular features include hypotension or clinical diagnosis of uncompensated shock (three signs of tachycardia, including capillary refill > 3s, reduced central pulse volume, or decreased consciousness). - Minor dermatologic, respiratory, cardiovascular, gastrointestinal, or laboratory features include less severe combinations of clinical features than the major categories.   Criteria for all levels of diagnostic certainty include:   - Acute and rapid onset (within 1 to 12 hours) AND - Involving multiple (>=2) organ systems   Level 1 (definite) diagnostic certainty requires >=1 major dermatological AND >=1 major cardiovascular AND/OR >=1 major respiratory criterion.  Level 2 (probable) diagnostic certainty requires:   - >=1 major cardiovascular AND >=1 major respiratory criterion OR - >=1 major cardiovascular OR respiratory criterion AND >=1 minor criterion involving an unrelated system OR - >=1 major dermatologic AND >=1 minor cardiovascular AND/OR minor respiratory criterion | Rüggeberg 2007 |
| **Guillain-Barre Syndrome (GBS)** | Neurologic adverse events following immunization (AEFIs) are among the most severe and most difficult to assess. The Brighton case definition for GBS was applied as follows:  Level 1 diagnostic certainty (definite) requires all six clinical findings below, while level 2 (probable) requires the first four findings and either 5 or 6.   1. Bilateral and flaccid weakness of the limbs AND 2. Decreased or absent deep tendon reflexes AND 3. Monophasic illness pattern AND interval between onset and nadir of weakness between 12 hours to 28 days AND subsequent clinical plateau AND 4. Absence of an identified alternative diagnosis for weakness AND 5. Cytoalbuminologic dissociation (i.e., elevation of CSF protein level above lab normal AND CSF total WBC count <50 cells/microL) AND 6. Electrophysiologic findings consistent with GBS. Additional criteria for the Fischer syndrome (FS) variant are provided as well. | Sejvar 2011 |
| **Intracranial or Intraabdominal Thrombosis With Thrombocytopenia Syndrome (TTS)** | Several cases of unusual thrombotic events and thrombocytopenia have developed after vaccination with the recombinant adenoviral vector encoding the spike protein antigen of severe acute respiratory syndrome coronavirus 2 (SARS-CoV-2) (ChAdOx1 nCov-19, AstraZeneca) (Greinacher 2021). More data were needed on the pathogenesis of this unusual clotting disorder. While a standard case definition for TTS does not yet exist, an interim definition from the Brighton Collaboration clarifies that the condition involves co-occurring thrombosis and new onset thrombocytopenia. Cases were independently verified for presence of thrombosis and thrombocytopenia. To be considered a TTS positive case, both the thrombosis and thrombocytopenia criteria were required to have been met:   - Thrombocytopenia - The Brighton Collaboration case definition for thrombocytopenia (7) is quite simple: a platelet count of less than 150,000/ul. A smear should also be evaluated to rule out platelet clumping. - Thrombosis - Newer versions of the Brighton Draft TTS standard (since November 11, 2021) include MI, PE, stroke, and extremity DVT for thrombosis sites. Earlier definitions used only venous intracerebral and intrabdominal thrombosis confirmed by imaging, surgery (thrombectomy), or pathology. The later of these definitions can be referred to as TTS at unusual sites, and that is the definition we applied in this study. For a positive determination to be made, an imaging report describing evidence of either intracranial or intra-abdominal venous thrombosis was required. Imaging modalities most used for evidence were: - Computed tomography (CT scan–contrast/angiography) - Magnetic resonance imaging or angiography (MR/MRA/MRV) - Ultrasound–Doppler - Conventional angiography/digital subtraction angiography - Surgical report (in cases of thrombectomy)   If the imaging report was not found because it was part of an earlier encounter involving patient transfer, a clinician note quoting the content of a recent positive imaging report could also serve as acceptable evidence. | Brighton Collaboration 2021 |
| **Febrile Seizure** | There is no Brighton Collaboration definition of febrile seizure. However, there are ones entitled “Fever as an adverse event following immunization: Brighton case definition” and “Generalized convulsive seizure as an adverse event following immunization: case definition and guidelines for data collection, analysis, and presentation.” This fever case definition defines fever as the presence of a temperature greater than or equal to 38 degrees Celsius (100.4 degrees Fahrenheit). The definition defends using the same 38-degree cutoff independent of the location or method of measurement (e.g., oral, aural, temporal, axial). Recorded temperature measurements in observations or notes were required to meet this criterion.  The types of seizure activity included were based on those seen in febrile seizure activity for children five years old or less. If there was evidence of positive fever with evidence of concurrent convulsions (generalized clonic or generalized tonic-clonic seizures) seen and described by a clinician in clinical notes or observations, then the diagnosis was confirmed as definite. If the fever criterion was met, but the seizure activity was second-hand (parent, transport) yet credible because it contained sufficient detail, then the determination was probable. | Marcy 2004; Bonohoeffer 2004 |
